# Low-flow vascular malformations without arteriovenous shunting of the central nervous system: a pictorial review

**DOI:** 10.1186/s13244-026-02209-4

**Published:** 2026-03-03

**Authors:** Lorena Nicolosi, Francesco Tiralongo, Corrado Inì, Daniele Grippaldi, Pietro Valerio Foti, Emanuele David, Cristina Mosconi, Stefania Tamburrini, Davide Giuseppe Castiglione, Giuseppe Messina, Rosita Comune, Roberto Minici, Stefano Palmucci, Antonio Basile

**Affiliations:** 1 Department of Medical Surgical Sciences and Advanced Technologies “GF Ingrassia”, Radiology Unit 1, University Hospital Policlinico “G. Rodolico-San Marco”, Catania, Italy; 2 Department of Medical Surgical Sciences and Advanced Technologies “GF Ingrassia”, UOSD IPTRA (Pulmonary and Advanced Radiological Techniques Unit University Hospital Policlinico “G. Rodolico-San Marco”, Catania, Italy; 3 Department of Radiology, IRCCS Azienda Ospedaliero Universitaria Di Bologna, Bologna, Italy; 4 Department of Radiology, Ospedale del Mare-ASLNa1 Centro, Napoli, Italy; 5 Radiology Unit, Department of Experimental and Clinical Medicine, University Hospital Mater Domini, Magna Graecia University of Catanzaro, Catanzaro, Italy

**Keywords:** Vascular malformations, Developmental venous anomaly, Cavernous hemangioma, Central nervous system, Magnetic resonance imaging

## Abstract

**Abstract:**

This review aims to provide a comprehensive pictorial review of low-flow vascular malformations (LFVMs) of the central nervous system (CNS) without arteriovenous shunting, focusing on their epidemiology, pathophysiology, imaging features, and associations with other vascular anomalies. LFVMs - developmental venous anomalies (DVAs), cavernous malformations (CMs), brain capillary telangiectasias (BCTs), and sinus pericranii (SP) - are typically benign and incidental but may cause symptoms or hemorrhage. Differentiating LFVMs from neoplastic, inflammatory, or high-flow vascular lesions is critical to avoid misdiagnosis and inappropriate treatment. MRI is the reference technique. DVAs show a “caput medusae” venous pattern; CMs have a mulberry-like core with a complete hemosiderin rim on T2*/SWI; BCTs are often occult on routine MRI but may display brush-like enhancement and subtle SWI hypointensity; SP consists of an extracranial venous mass communicating with a dural sinus through a transosseous vein. Familiarity with the imaging spectrum and typical associations of CNS LFVMs enables confident diagnosis and helps avoid unnecessary invasive procedures.

**Critical relevance statement:**

By illustrating key imaging features of low-flow CNS vascular malformations, this article critically addresses frequent diagnostic pitfalls. It advances radiological practice by guiding differentiation from neoplastic or high-flow lesions and improving multidisciplinary patient care.

**Key Points:**

- LFVMs (DVAs, CMs, capillary telangiectasia, SP) are frequently incidental but may cause hemorrhage, seizures, or neurological deficits.
- DVAs are typically benign drainage variants; hemodynamic congestion on perfusion weighted imaging explains occasional symptoms and the frequent association with acquired CMs.
- CMS presents as “mulberry-shaped” lesions with a hemosiderin rim on SWI sequences, reflecting microhemorrhages and the absence of intervening brain parenchyma.
- Capillary telangiectasia most often occurs in the pons; recognition of the characteristic SWI hypointensity with faint enhancement prevents misdiagnosis as a neoplasm or ischemia.
- AP shows trans‑osseous venous channels connecting dural sinuses to epicranial varices; CT characterizes bony defects, and MRI depicts venous communication.

**Graphical Abstract:**

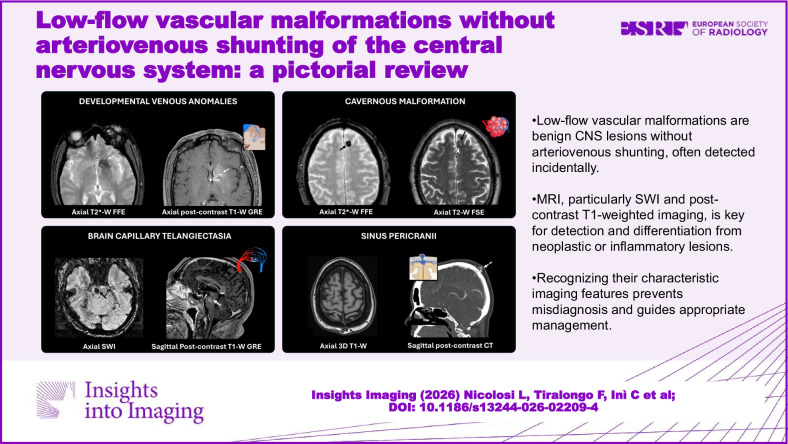

## Introduction

**Fig. 1:**
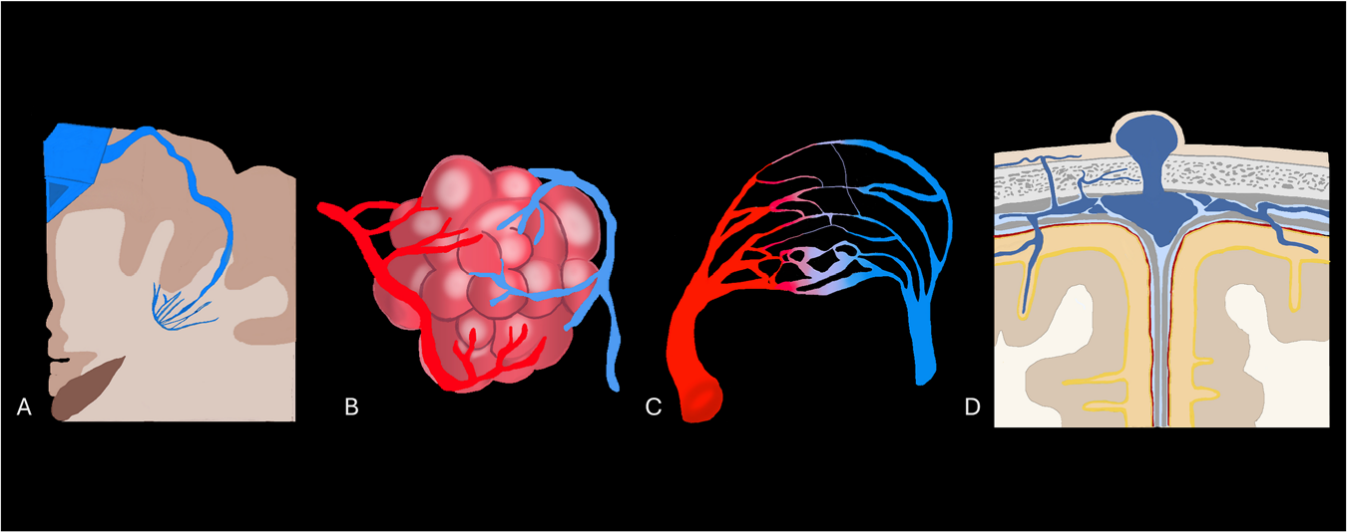
Illustrations of developmental venous anomaly (DVA) (**A**), CM (**B**), capillary telangiectasia (TC) (**C**), and SP (**D**)

Low-flow vascular malformations (LFVMs) are uncommon brain vascular malformations that generally present with an indolent course but may acutely manifest with hemorrhage, seizures, or neurological deficits [1]. This group - distinct from high‑flow arteriovenous shunting lesions - encompasses congenital and acquired malformations affecting venous and capillary structures: developmental venous anomalies (DVAs), cavernous malformations (CMs), brain capillary telangiectasia (BCTs), and sinus pericranii (SP) (Fig. 1) [1]. Magnetic resonance imaging (MRI) has enhanced the detection of both isolated and combined lesions, known as “mixed vascular malformations,” and prompted a renewed interest in their pathophysiology and clinical management [2–6]. CT, although less sensitive for subtle lesions, remains valuable for calcifications and acute hemorrhage [1, 6–8].

This pictorial review provides a comprehensive overview of the epidemiology, pathogenesis, clinical presentation, and multimodality imaging features of LFVMs without arteriovenous shunting. Special emphasis is placed on MRI and CT features that facilitate accurate diagnosis, differentiation from other intracranial pathologies, and recognition of associated vascular anomalies.

## Imaging protocol

The MRI protocol performed at our institution, using a superconducting 1.5-T system (SIGNA Explorer, GE Healthcare) and a 16-channel high-resolution neurovascular coil, is summarized in Table 1.

**Table 1:** MRI brain protocol at our institution

	TR/TE (ms)	Flip angle	Slice thickness (mm)	Interslice gap (mm)	Bandwidth (kHz)	Field of view (cm)	Matrix	No. of averages	No. of images	Frequency direction	Acquisition time	B-value (s/mm^2^)
Sagittal T2-W FLAIR FS 3D TSE	5000/120	90°	1.30	-	20.83	26.6	200 × 200	1.00	120	Superior/inferior	5.28 min	-
Axial T2-W 2D FSE	7228/102	90°	4.00	0.5	35.71	25.0	384 × 320	2.00	35	Anterior/posterior	2.32 min	-
Axial T2-W FLAIR 2D FSE	9333/120	90°	4.00	0.5	27.78	25.0	320 × 224	1.00	36	Anterior/posterior	2.39 min	-
Axial SWI 3D	64/48	15°	1,10	-	41.67	24.00	320 × 256	-	132	Anterior/posterior	4.41 min	-
Axial DWI SE EPI	7128/80.8	90°	4.00	0.5	250	25.0	96 × 128	3.00	35	Right/ left	1.26 min	0-1000
Axial T1-W 3D GRE	7.3/2.7	12°	1.30	-	27.78	25.6	200 × 200	1.00	118	Anterior/popsterior	4.07 min	-
Axial T1-W 2D SE	540/11	71°	4.00	1.0	25.00	25.0	288 × 192	2.00	32	Anterior/posterior	3.43 min	-
Sagittal T1-W 3D GRE + c	7.3/ 2.7	12°	1.30	-	27.78	25.6	200 × 200	1.00	118	Anterior/posterior	4.07 min	-
Axial T1-W 2D SE + c	540/ 11	71°	4.00	1.0	25.00	25.00	288 × 192	2.00	32	Anterior/posterior	3.43 min	-

We consider it essential that the brain MRI protocol systematically includes susceptibility-weighted imaging (SWI) and post-contrast T1-weighted sequences, as these acquisitions are critical for optimal detection and characterization of LFVMs.

SWI leverages differences in magnetic susceptibility between diamagnetic (calcium) and paramagnetic (deoxyhemoglobin, hemosiderin) sources, enabling quantitative susceptibility mapping (QSM) to distinguish calcific from hemorrhagic material [8]. Post-contrast 3D T1‑weighted images with thin, contiguous slices are crucial for depicting small, enhancing lesions (often a few millimeters (mm)) such as capillary telangiectasia [8, 9].

## Developmental venous anomaly (DVAs)

**Fig. 2:**
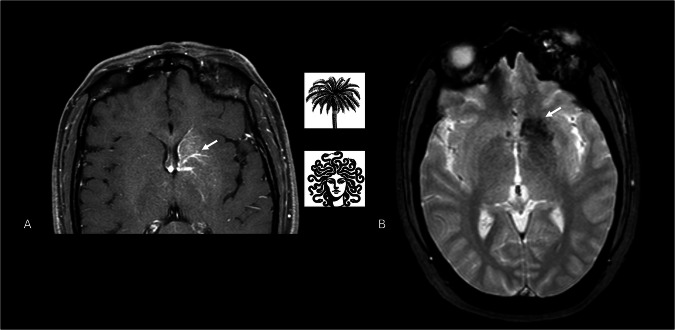
Axial T1-W post-contrast image shows a typical periventricular “caput medusae” or “palm-tree” appearance of the DVA (white arrow) that drains into the subependymal veins within the frontal horn of the left ventricle (**A**). Axial T2* GRE sequence of the same case depicts classic blooming artifacts of the DVA (white arrow) due to the hemosiderin content consequent to blood stasis within the venous anomaly (**B**)

DVAs, formerly known as venous angiomas, are understood as extreme anatomical variations of normal medullary venous drainage rather than true malformations [10]. They consist of radially arranged medullary veins that converge into a transcortical or subependymal collector vein, creating the classic “caput medusae” pattern (Fig. 2) [4, 11].

The reported prevalence has increased with the widespread adoption of MRI, from 2.6% in early series to approximately 7% in recent studies [4, 12].

DVAs occur predominantly in the supratentorial compartment, with the frontal lobe most affected (35%), followed by the parietal lobe (15%) and basal ganglia (4%) [13]. Venous drainage is directed toward the superficial system in about 70% of cases, the deep system in 20%, and both systems in 10% [10, 14]. DVAs are more often seen as isolated cases. However, in 7.56% to 16% of cases, they appear as multiple anomalies, with some cases having up to four concurrent irregularities, especially in syndromic conditions. These include syndromic environments such as cervicofacial venous metameric syndrome (CVMS), blue rubber bleb nevus syndrome, and constitutional mismatch repair deficiency (CMRRD) [15–17].

**Fig. 3:**
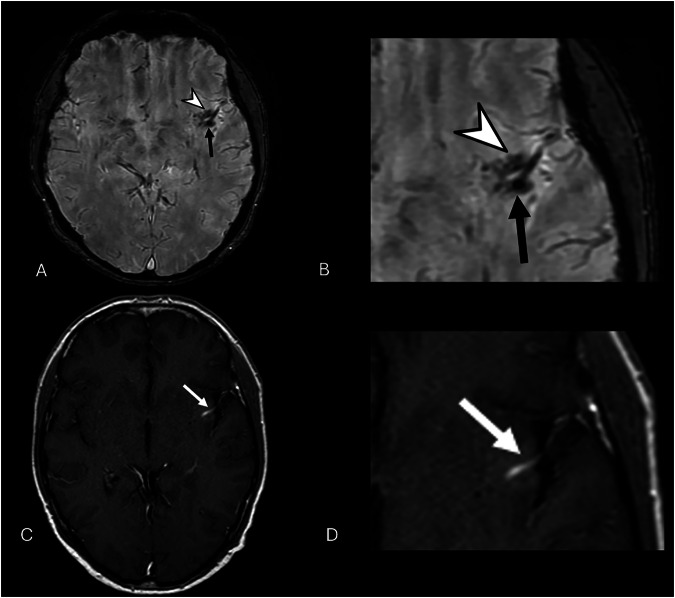
Axial 3D SWI image shows a blurred hypointensity within the insular cortex (arrowhead), associated with a small, punctiform, and well-defined hypointensity (thick black arrow) (**A**, **B**). Axial post-contrast T1-W image confirms the presence of a DVA, characterized by linear enhancement (white arrow) and superficial drainage (**C**, **D**). The absent enhancement on post-contrast images of the punctiform lesion, visible exclusively on the SWI image, indicates the concomitant presence of a type IV CM

**Fig. 4:**
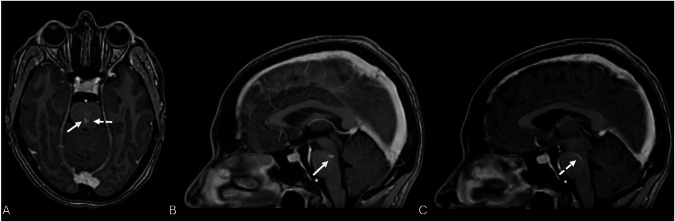
Axial and sagittal 3D T1-W GRE post-gadolinium administration reveal two different structures, one punctate and focal enhancement (white arrows, **A**, **B**) and nearby a linear enhancement (dotted arrow) which has a continued course through mid-pons, also visible on sagittal imaging (dotted arrow, **A**, **C**), till draining into vessels of the interpeduncular cistern. This is the case of a capillary telangiectasia (white arrow) associated with a pontine DVA (dotted arrow)

DVAs are frequently associated with other vascular anomalies. CMs are the most common, occurring in 3.4–13.3% of cases [13], likely due to chronic venous hypertension promoting microhemorrhage and angiogenesis (Fig. 3) [4, 18, 19]. Capillary telangiectasias are less frequent but occur more often in posterior fossa DVAs (Fig. 4) [20, 21]. Other reported associations include SP [22], cerebral varices [23], and, rarely, arteriovenous malformations (AVMs) [24].

DVAs have been reported more often in patients with multiple sclerosis than in the general population, possibly due to perivenular inflammation facilitated by altered venous drainage (Fig. S1) [25]. They may also coexist with cortical malformations such as pachygyria, focal cortical dysplasia, and polymicrogyria, suggesting a shared developmental origin [16, 26].

**Fig. 5:**
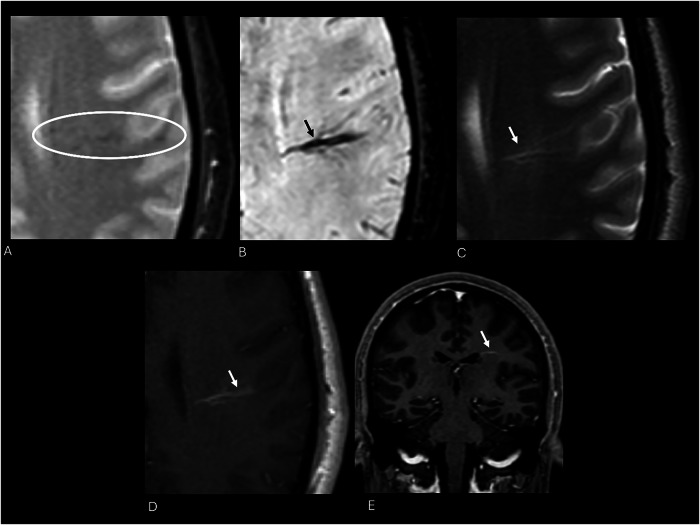
MRI performed for a 40-year-old male patient with status epilepticus. T2* image displays a significantly noticeable hypointensity in the left frontal lobe (white circle) (**A**), which becomes evident on SWI as a linear flow-void signal (black arrow) (**B**). On axial T2 turbo spin‑echo (TSE), the anomaly shows a branched vascular structure with high signal intensity, with subcortical involvement (white arrow) (**C**). Post gadolinium axial 2D T1-W spin‑echo (SE) (**D**) and coronal 3D T1-W GRE (**E**) display the characteristic linear enhancement of the ectatic collector vein draining into subependymal veins of a DVA

Because DVAs often serve as the only venous drainage for the affected area, thrombosis or surgical interruption can lead to venous infarction or hemorrhage [27]. Most are asymptomatic and found incidentally; only 2% become symptomatic, usually due to hemorrhage or ischemia [26]. When symptoms occur, they are nonspecific and may include headache, seizures, dizziness, or altered consciousness [5, 26]. Rarely, DVAs near the aqueduct can cause obstructive hydrocephalus (Fig. 5) [16].

Computer Tomography (CT) is infrequently effective in providing a confident diagnosis of DVAs.

On non-contrast CT, DVAs are generally inapparent unless complicated by hemorrhage or thrombosis [10], [14]. Contrast-enhanced CT may reveal enhancing linear structures within the white matter, but posterior fossa lesions are often obscured by beam-hardening artifacts [10].

MRI is the modality of choice, with susceptibility-weighted imaging (SWI) demonstrating the highest sensitivity (86%) and specificity (93%) [11, 28, 29].

On SWI, a high-resolution three-dimensional gradient echo (GRE) sequence, DVAs are usually visible as cortical or subependymal linear flow voids caused by the dilated medullary vessels, with the typical “umbrella appearance” or of the collector vein due to paramagnetic deoxyhemoglobin content (Fig. 5) [11, 28].

**Fig. 6:**
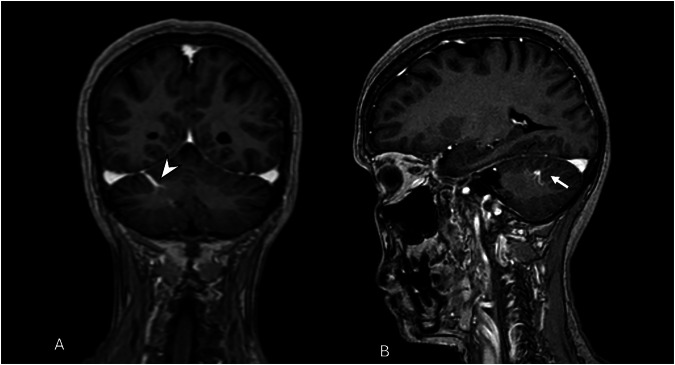
Rare case of infratentorial DVA. Coronal (**A**) and sagittal (**B**) 3D T1-W GRE images show linear contrast enhancement of the right cerebellar hemisphere, typical of a DVA emissary vein draining into the right transverse sinus (arrowhead). Caput medusae sign (**B**) is characterized by multiple anomalous medullary veins draining into a larger collecting vein (white arrow)

T1-weighted spin-echo (SE) images can show a normal brain parenchyma signal. Post-contrast T1 SE sequence usually demonstrates the presence of a linear cortical or subependymal enhancement of the DVA, with an umbrella appearance, or so-called “caput medusae”, draining in a superficial or deep vein collector (Fig. 6) [30].

Parenchymal signal changes are seen in approximately 10% of cases, usually as T2/fluid‑attenuated inversion recovery (FLAIR) hyperintensity within the drainage territory, reflecting chronic venous congestion, gliosis, or white matter changes [26, 28, 31]. Perfusion MRI may reveal increased regional cerebral blood volume and prolonged mean transit time, occasionally accompanied by mildly elevated cerebral blood flow due to compensatory vasodilation [18].

The main differentials include transitional venous anomalies (TVAs) and AVMs. TVAs resemble DVAs morphologically but demonstrate arteriovenous shunting on perfusion imaging and early venous/parenchymal filling on digital subtraction angiography (DSA). They may represent sequelae of venous thrombosis with collateralization [32]. Arterial spin labeling (ASL) can help detect shunting and avoid unnecessary DSA in benign variants [33]. AVMs differ by the presence of nidus, arterial feeders, and high-flow hemodynamics [14].

## Cavernous malformations (CMs)

CMs represent 5–15% of all cerebral vascular malformations, making them the second most common low-flow venous anomaly after DVAs [34]. Histologically, they are clusters of endothelium-lined vascular sinusoids devoid of intervening brain parenchyma and surrounded by a collagenous wall. Their slow-flow nature predisposes them to recurrent microthrombosis, recanalization, and calcification. Disruption of the blood–brain barrier (BBB) promotes erythrocyte extravasation and hemosiderin deposition [35–37].

The prevalence in the general population ranges from 0.1% to 0.8%, with a median age at diagnosis of 37 years; however, lesions can present at any age [36–38]. CMs may occur sporadically or as part of familial forms. Familial CMs, associated with loss-of-function mutations in CCM1, CCM2, or CCM3 genes, are often multiple [39]. Radiation-induced CMs are also well recognized, typically developing years after exposure [34, 39].

DVAs are the most frequent vascular anomaly associated with sporadic CMs, suggesting that altered venous drainage may contribute to CM development [40]. Most CMs (95%) are incidentally discovered [41]. Symptomatic lesions typically present with seizures (40–80%), focal neurological deficits (20–40%), intracerebral hemorrhage (25–40%), or headache (10–30%) [38, 42]. The clinical presentation is influenced by lesion location: supratentorial frontal and temporal CMs are more prone to seizures, whereas brainstem lesions more often produce cranial nerve palsies and long-tract signs, with worse long-term outcomes [35, 43, 44]. Annual hemorrhage risk is 0.7% for incidentally detected lesions, increasing to 6% for brainstem CMs, with higher recurrence rates after an initial bleed [44].

On CT, acute hemorrhage and calcifications are well-detectable, but angiography is typically negative due to the low-flow nature of the lesion [45, 46]. MRI is the modality of choice.

**Fig. 7:**
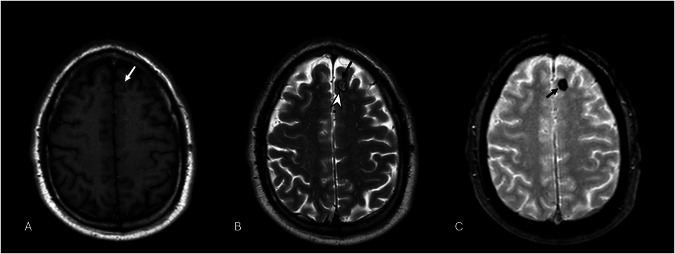
Axial 2D T1-W SE shows a blurred spot with an isointense core and hypointense rim in the left frontal lobe (white arrow) (**A**). On T2-W FSE images, the lesion shows a “pop-corn” appearance with central high signal intensity (black arrow) and a hypointense hemosiderin pad (arrowhead) (**B**). Moreover, it presents susceptibility magnetic artifacts on T2*-W FFE, appearing as a hypointense lesion, typical of type II CM (**C**)

CCMs typically present as round-shaped multilobulated lesions, with a characteristic “popcorn” or “mulberry” appearance [46]. They typically exhibit a reticulated appearance with mixed T1/T2 signal, reflecting different-aged blood products resulting from recurrent microhemorrhages and thrombosis. The presence of paramagnetic hemosiderin and ferritin deposits, following intralesional hemorrhages, induces magnetic susceptibility and blooming artifacts that manifest as a hypointense rim surrounding these vascular lesions on T2*GRE and SWI images, thereby increasing the sensitivity to detect even multiple CCMs, which are typical of familial CCMs (Fig. 7) [6], [47]. They may show a “feeding-vessel sign” or mass effect in hemorrhagic lesions, but usually lack vasogenic edema [47]. Moreover, MRI also monitors lesion growth and the development of new CMs during follow-up. Post-contrast T1 imaging usually does not show vascular enhancement of CMs, but highlights them when combined with DVA or capillary telangiectasias [47]. SWI and QSM have enhanced the detection of small or atypical lesions, allowing for the assessment of susceptibility changes over time [38, 48, 49]. Perfusion MRI may show mildly increased permeability.

Atypical lesions without a complete hemosiderin rim can mimic neoplasms, especially when surrounded by edema or exhibit irregular enhancement.

**Fig. 8:**
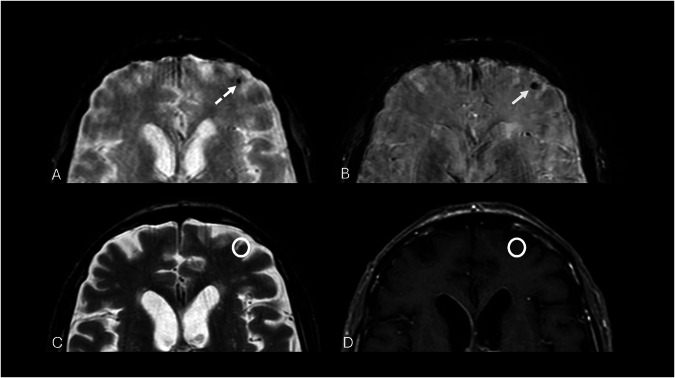
Axial 2D T2* GRE sequence presents a small hypointense dot on the left subcortical frontal lobe (dotted arrow) (**A**), that seems to be emphasized in 3D SWI imaging (white arrow) (**B**). Axial 2D T2-W FSE (**C**) and T1-W SE (**D**) images make this lesion barely visible (white circles), as indicated on type IV CMs according to Zabramski classification

Zabramski et al classified CMs into four MRI types (Fig. 8 and Table 2) [38, 43, 50].

**Table 2:** Zabramski's classification of CMs

CMs type	W	MRI signal	Pathological characteristics
Type I	T1: T2:	Hyperintense core Hyper- or hypointense core with hypointense rim	Subacute hemorrhage with hemosiderin rim
Type II	T1: T2: T2*:	Popcorn lesion with heterogeneous signal intensity centrally Popcorn lesion with heterogeneous signal intensity centrally Hypointense lesion with blooming	Irregular areas of hemorrhage and thrombosis at various evolutive stages
Type III	T1: T2: T2*:	Iso- hypointense core Hypointense core Hypointense lesion with blooming	Chronic hemorrhage with hemosiderin foci within and around the lesion
Type IV	T1: T2: T2*:	Isointense to brain parenchyma Isointense to brain parenchyma Hypointense spot lesion	Punctate microhemorrhages

**Fig. 9:**
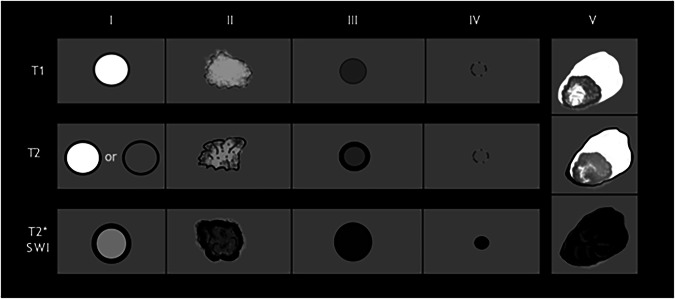
MRI illustration of the signal intensity of CMs according to Zabramski classification on T1, T2, and T2* weighted images. Type 5 represents a new entity characterized by CM within a brain hemorrhage

In 2015, Nikoubashman et al described type 5 CM, which is characterized by CMs presenting with an evident extralesional hemorrhage, characterized by heterogeneous signal intensity corresponding to the age of blood products and hypointensity on SWI or T2* GRE sequences (Fig. 9 and Table 3) [51].

**Table 3:** Summarized features of low-flow vascular anomalies of the central nervous system

LOW-FLOW VASCULAR ANOMALIES	Typical localization	Clinical implications	Imaging features
Developmental venous anomaly (DVA)	• Sovratentorial (frontal lobe, parietal lobe, basal ganglia) • Infratentorial (cerebellum, brain stem, spine).	• Benign and asymptomatic • Hemorrhages, venous infarction, hydrocephalus	**MRI** • T1-W + c: “caput medusae”, draining in a superficial or deep vein collector • SWI or T2* GRE: magnetic susceptibility artifacts due to deoxyhemoglobin content of the anomalous medullary veins.
Cerebral cavernous malformation (CCM)	• Sovratentorial (frontal and temporal lobes); • Infratentorial (brainstem and spinal cord).	• Seizures • Focal neurological impairments • Intraparenchymal hemorrhage • Headaches	**MRI** • T2-W: high-signal intensity with a “popcorn” or “mulberry” aspect. • T1-W + c: no contrast enhancement • SWI or T2* GRE: blooming artifacts due to hemosiderin components.
Capillary telangiectasia (TC)	• Pons; • Cerebral hemispheres; • Gray matter nuclei, cerebellum, midbrain, spinal cord.	• Asymptomatic • Seizures, vertigo, migraines, cranial nerve palsies, visual impairments, cerebellar syndromes, or myelopathy	**MRI** • T2-W: iso- or hyperintense signal • SWI or T2* GRE: central signal drop • T1-W + c: spotty or linear enhancement
SP (SP)	• Median (near the falx cerebri in the frontal region); • Paramedian (parietal region); • Lateral (temporal region).	• Palpable soft-tissue mass, skin dyschromia • Focal tenderness, pain • Vertigo, general headache, nausea	**CTA** • Soft-tissue-enhancing mass with sagittal sinus connection and associated scalp erosion **MRI** • T1-W: isointense with signal voids • T2-W: hyperintense signal • 2D-TOF: visualize the venous connection within the scalp • T1-W + c: communications between the dural sinus and SP through diploic veins

Differential diagnoses include AVMs, hypertensive or amyloid-related cerebral microbleeds, hemorrhagic metastases, and radiation-induced telangiectasias [52–57]. Mixed malformations are common; up to 30% of CMs are associated with DVAs [56–58].

## Brain capillary telangiectasias (BCTs)

BCTs are uncommon LFVMs, histologically composed of clusters of dilated capillaries separated by normal brain parenchyma and lacking muscular or elastic components [59–61]. First described in early autopsy studies, they were recognized as a distinct vascular anomaly in the second half of the 20th century following the advent of modern neuroimaging [59].

BCTs represent 16–20% of all cerebral vascular malformations in autopsy series, but their true prevalence is underestimated because most remain clinically silent [60].

The median age of onset is 47 years, with a small prevalence in the female population (approximately 55% of instances) [59].

They are most frequently located in the pons (60–80%), followed by the medulla, midbrain, and, less commonly, the cerebellum or cerebral hemispheres [60, 62]. Lesions are usually solitary but may be multiple in patients with hereditary hemorrhagic telangiectasia or associated with other vascular anomalies such as CMs and DVAs [63–65].

Capillary telangiectasia generally follows an indolent and asymptomatic course, except for large ones, which are most often discovered after developing symptoms. Some of the clinical implications observed in non-hereditary BCTs are focal or generalized seizures, vertigo, migraines, cranial nerve palsies, visual impairments, and occasionally cerebellar syndromes or myelopathy [62, 63].

The physiological evolution of symptomatic relevant telangiectasias might be hemorrhages, diffuse parenchymal lesions, and/or ischemic necrosis. Rarely, hemorrhage might manifest because of venous hypertension, causing the rupture of weak and anomalous capillaries [64], particularly in hereditary hemorrhagic telangiectasia (HHT), also known as Rendu-Osler-Weber disease [65, 66]. In this case, the coexistence with other vascular anomalies, such as CMs or DVAs, must be taken into consideration for the diagnosis [67]. BCTs are blurred and small capillary alterations, hardly visible on unenhanced CT (except for the presence of rare calcifications) or DSA. Post-contrast CT may show a fine area of enhancement in a typical pontine region [68].

**Fig. 10:**
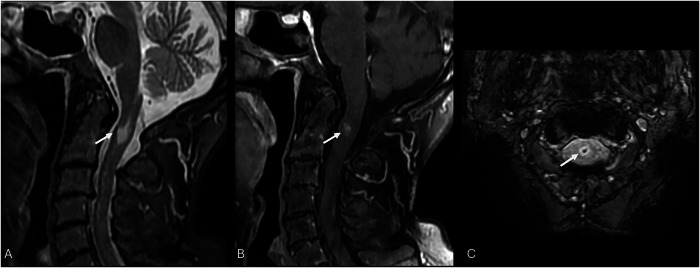
Cervical spine MRI scans show an intradural mass at the C2 level. The mass shows high-signal intensity on sagittal T2-W FS STIR image (white arrow, **A**), homogeneous and strong enhancement (white arrow, **B**), and blooming artifacts on axial SWI (white arrow, **C**). These are incidental findings of a rare spinal cord capillary telangiectasia

**Fig. 11:**
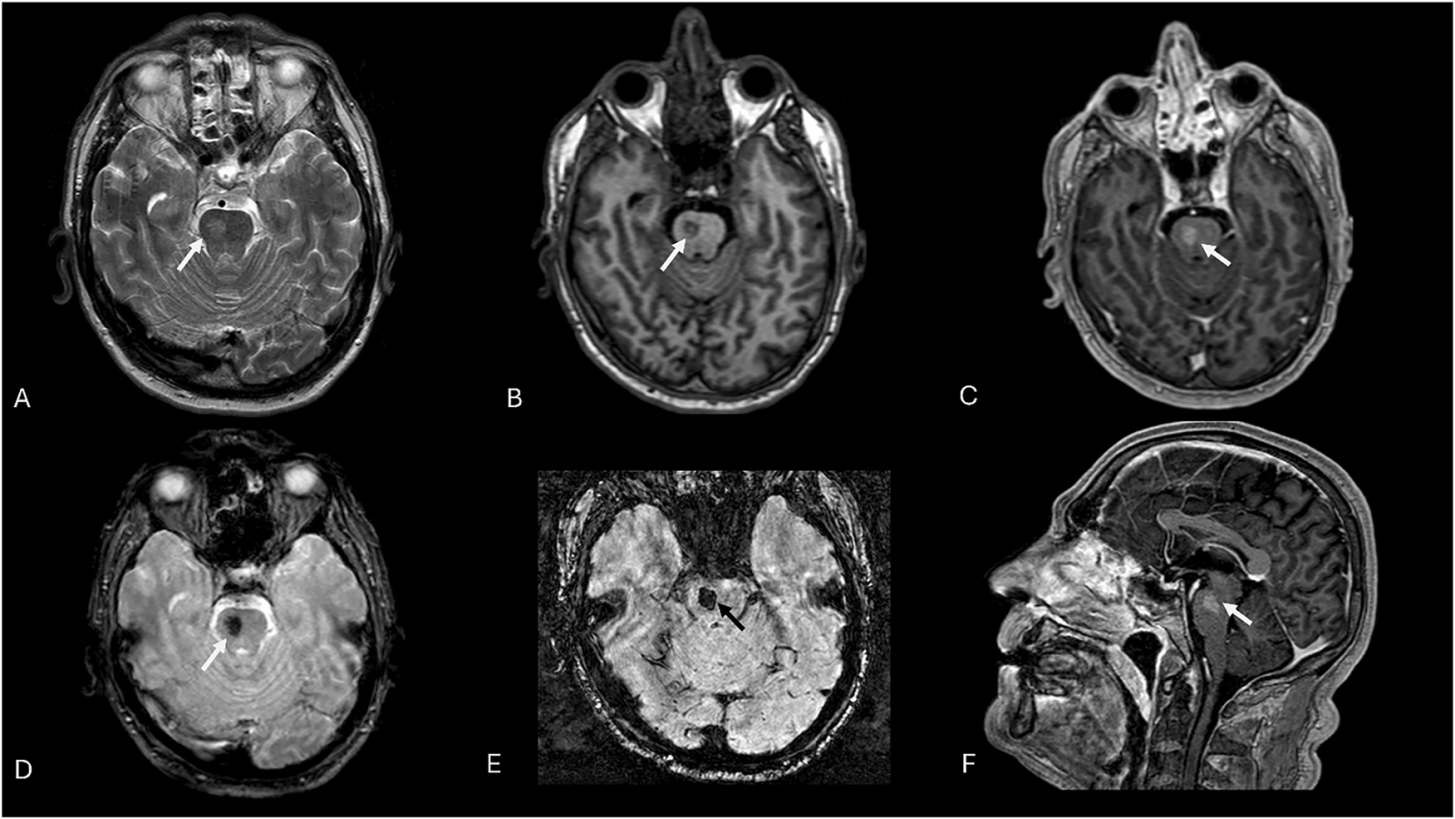
Axial 2D T2-W TSE shows a blurred hyperintensity on the right paramedian pons (white arrow, **A**), which appears hypointense on pre-contrast axial 3D T1-W GRE (white arrow, **B**) and with pronounced enhancement on axial (**C**) and sagittal (**F**) post-contrast 3D T1-W GRE images (white arrows). Axial 2D T2*-GRE (**D**) and 3D-SWI (**E**) show characteristic magnetic susceptibility artifacts of the pontine lesion. Imaging features and typical localization are typical markers of capillary telangiectasia

Even at unenhanced MRI, on T2 or FLAIR sequences, telangiectasias may be unrevealed or exhibit signal hyperintensity without vasogenic edema or a mass effect. SWI and T2*-GRE reveal a diffuse or central signal drop due to deoxyhemoglobin deposits, and a lack of diffusion restriction on DWI helps in differentiating BCTs from subacute ischemic stroke or neoplasms [29, 66, 67]. Post-contrast T1-WI has increased the premortem diagnosis of such anomalies, sometimes revealing the unique sequences that make them visible; they manifest as spotty or linear enhancement, typically less than 2 cm in diameter, and are often located on the pons, where the differential diagnosis with CCM might be blurred (Figs. 10 and 11 and Table 3).

Generally, differential diagnosis can be challenging and may encompass CM. Given the higher risk of hemorrhagic complications associated with CCM, it should be considered the most probable diagnosis when present [1]. Additionally, CCM is characterized by gliosis, fibrosis, and hemosiderosis of the nearby parenchyma, whereas BCT is typically surrounded by normal parenchyma [64]. MRI investigation is sufficient to establish a confident diagnosis of BCT and to rule out inflammatory diseases (e.g., acute demyelination), neoplastic processes (e.g., astrocytoma, metastases, lymphoma), and subacute ischemia. When atypical characteristics are present, the absence of a mass effect or vasogenic edema suggests vascular anomalies rather than gliomas or metastasis [67, 68].

## Sinus pericranii (SP)

SP is a rare, low-flow venous malformation first described by Hecker in 1845 and more precisely defined by Stromeyer in 1850 [69]. It is characterized by an abnormal communication between intracranial dural sinuses and epicranial veins through transosseous emissary veins, which may become varicose under conditions of elevated intracranial pressure. These emissary veins emerge from the periosteum and may form a soft scalp mass that, over time, can cause skull erosion [70].

The etiology of SP remains debated. A congenital origin is supported by frequent associations with other cerebral vascular malformations [70]. In contrast, an acquired form may result from increased intracranial pressure during embryogenesis, which reduces epicranial drainage into dural sinuses and leads to transosseous venous dilatation, or from direct traumatic injury to the emissary veins [71].

It can occur at any age, but pediatric onset peaks at approximately 62.5 months, with a slight prevalence in females (62%) [72]. SP most commonly involves the frontal region near the falx cerebri, draining into the superior sagittal sinus (40%), followed by the parietal (34%), occipital (23%), and temporal (4%) regions [72–74]. SP has been associated with syndromic disorders such as Noonan syndrome with bilateral craniosynostosis, Coffin–Siris syndrome, and 17q deletion syndrome, as well as non-syndromic cases with dural sinus hypoplasia, interventricular septal defect, aortic coarctation, and blepharophimosis [75].

SP often coexists with other LFVMs, supporting a congenital origin [76, 77]. Associations include DVAs, CMs, blue rubber bleb nevus syndrome (BRBNS), and tongue venous malformations, with over 20% of cases demonstrating multiple lesions [78]. Sherry et al reported a BRBNS case in which a large DVA drained into an extracranial SP via an enlarged varix [79].

While often asymptomatic, SP may present with headache, vertigo, nausea, focal scalp tenderness, or pain. Less common symptoms include bradycardia, bradypnea, seizures, and hearing loss [80, 81]. Potential complications include partial sinus thrombosis, traumatic intracranial hemorrhage, and intracranial infection [82]. Clinically, the lesion appears as a compressible scalp mass, occasionally with overlying skin discoloration or hyperemia (9.5%). Enlargement is often observed during the Valsalva maneuver, crying, or supine positioning, and may be accentuated by jugular vein compression [72].

Color Doppler ultrasound (CDUS) is often the first-line modality, revealing a compressible hypoechoic tubular structure within the subcutaneous tissue [73, 83].

Contrast-enhanced CT (CECT) typically shows an enhancing soft-tissue mass with adjacent skull erosion, which is > 3 mm more often in high-flow forms [76, 84]. Unenhanced CT can delineate defect size, shape, and margins, which are critical for endovascular planning [72]. CT angiography with early and delayed phases demonstrates early homogeneous venous enhancement connected to the superior sagittal sinus via transosseous collaterals. Partial thrombosis appears as a delayed filling defect [85]. Similar CT findings can also occur in reactive intravascular papillary endothelial hyperplasia (also known as Masson’s tumor) [86].

**Fig. 12:**
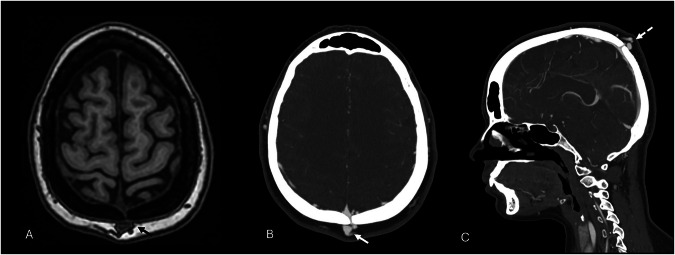
Axial 3D T1-W image shows some anomalous dilated vessels (black arrow) within scalp tissues (**A**). Axial and sagittal images from a CT with venous phase, demonstrate the anomalous connection between the subgaleal vein and the superior sagittal sinus (white continuous and dotted arrows) within a defect of the parietal bone (**B**, **C**)

MRI provides excellent soft-tissue characterization, confirming the epicranial varix and depicting associated anomalies such as dural sinus hypoplasia [72]. T1-weighted sequences show isointense or mixed signal with flow voids; T2-WI demonstrates hyperintensity relative to scalp tissue [86]. Post-contrast T1-weighted spin-echo images depict venous communications through diploic veins and transosseous channels, distinguishing SP from subepicranial varices or arteriovenous fistulas (Fig. 12) [70]. Thrombosis appears as heterogeneous, non-enhancing regions, but an evolving clot signal may complicate interpretation [76, 87]. Non-contrast 2D-time‑of‑flight (TOF) MR angiography can noninvasively demonstrate transosseous venous connections, though CT remains superior for evaluating osseous defects [76, 88]. Févre and Modec classified SP into three hemodynamic types, summarized in Table 4 [83, 89].

**Table 4:** Févre and Modec hemodynamic SP classification

	Févre and Modec SP classification
Type I	Closed-loop systems with bidirectional flow between the sinus and extracranial veins
Type II	Unidirectional drainage from the sinus to the peripheral veins
Type III	“Pseudo-SP”: abnormal communications between the sinus and an angioma or aneurysm with arterial components

Imaging is essential to confirm the diagnosis and exclude differential diagnoses such as meningocele, leptomeningeal cyst, encephalocele, arteriovenous fistula, and cavernous hemangioma [90].

## Conclusion

LFVMs form a clinically significant subset of cerebrovascular malformations. Although often incidental, they have non-trivial implications due to potential symptoms, increased risk of hemorrhage, and frequent coexistence as mixed malformations. MRI - particularly SWI (±QSM) and thin-slice post-contrast 3D T1 - underpins confident recognition, while CT complements this by detecting calcifications, acute hemorrhage, and bone defects. Early, accurate diagnosis reduces misclassification, guides surveillance vs intervention, and improves multidisciplinary care.

Understanding the full spectrum of LFVMs, their possible associations, and their imaging features is essential for radiologists, as these lesions, though frequently indolent, may have significant diagnostic and therapeutic implications.

## Supplementary information

ELECTRONIC SUPPLEMENTARY MATERIAL

## Data Availability

All data are available from the corresponding author. All data and materials are available at the Department of Medical Surgical Sciences and Advanced Technologies “GF Ingrassia”, Radiology Unit 1, University Hospital Policlinico “G. Rodolico-San Marco”, Catania, 95123, Italy; in particular, in our RIS-PACS system/archive.

## Abbreviations

BCT: Brain capillary telangiectasia
CM: Cavernous malformation
CT: Computed tomography
DSA: Digital subtraction angiography
DVA: Developmental venous anomaly
FLAIR: Fluid‑attenuated inversion recovery
GRE: Gradient echo
MRI: Magnetic resonance imaging
QSM: Quantitative susceptibility mapping
SE: Spin‑echo
SP: Sinus pericranii
SWI: Susceptibility‑weighted imaging
TSE: Turbo spin‑echo
