# Supplementary material for: Low-flow vascular malformations without arteriovenous shunting of the central nervous system: a pictorial review
Source: Insights Imaging. 2026 Mar 3;17:60. doi: 10.1186/s13244-026-02209-4 (PMC12957764; doi:10.1186/s13244-026-02209-4)

Low-flow vascular malformations without arteriovenous shunting of the central nervous system: a pictorial review

ELECTRONIC SUPPLEMENTARY MATERIAL

Figure S1. Axial 3D SWI and miP SWI reconstruction show pontine DVA with median collecting vein (white arrows) (A,B) associated with adjacent multiple sclerosis plaques (arrowheads) (C).

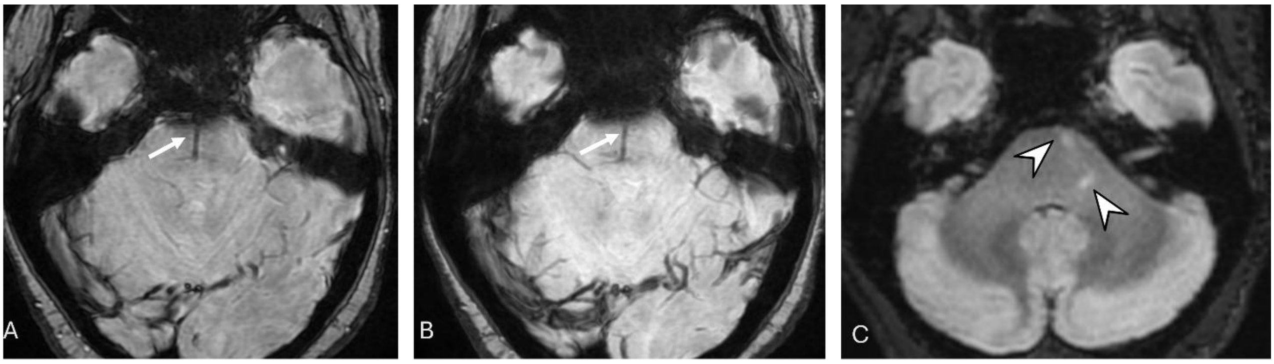

Supplement: Supplementary file 1 — ELECTRONIC SUPPLEMENTARY MATERIAL [file 13244_2026_2209_MOESM1_ESM.pdf]
